# Research on Chengdu Ma Goat Recognition Based on Computer Vison

**DOI:** 10.3390/ani12141746

**Published:** 2022-07-07

**Authors:** Jingyu Pu, Chengjun Yu, Xiaoyan Chen, Yu Zhang, Xiao Yang, Jun Li

**Affiliations:** 1 College of Information Engineering, Sichuan Agricultural University, Ya’an 625000, China; pujingyu@stu.sicau.edu.cn (J.P.); 202005807@stu.sicau.edu.cn (C.Y.); 201902158@stu.sicau.edu.cn (Y.Z.); 202005537@stu.sicau.edu.cn (X.Y.); lijun@sicau.edu.cn (J.L.); 2 Sichuan Key Laboratory of Agricultural Information Engineering, Ya’an 625000, China

**Keywords:** precision livestock farming, deep learning, computer vision, object detection, self-supervised learning, Chengdu ma goat

## Abstract

**Simple Summary:**

Accurate and efficient automatic individual recognition of livestock such as goats is one of the key tasks to achieve smart farming. However, this task is challenging due to factors such as the dense distribution of livestock in a barn. To accurately recognize Chengdu ma goats, a high-quality sheep breed with local characteristics, we construct a Chengdu ma goat dataset containing nearly one thousand images with manual annotation information and propose a method for the automatic individual recognition of Chengdu ma goats using a computer. Experiments demonstrate the usability of our method to accurately recognize Chengdu ma goats under extreme conditions such as dense target distribution and drastic differences in scale. It is worth noting that the method is scalable, which enhances the network’s representation ability for data by learning the structural information of goats’ head area. The location and category information obtained by our automatic individual recognition method is the basis for realizing a series of subsequent intelligent breeding tasks, such as precision feeding based on age and sex. Our method provides a new idea for automatic livestock recognition and can be extended to be applied to additional types of livestock.

**Abstract:**

The Chengdu ma goat is an excellent local breed in China. As one of the breeds listed in the National List of Livestock and Poultry Genetic Resources Protection, the protection of its germplasm resources is particularly important. However, the existing breeding and protection methods for them are relatively simple, due to the weak technical force and lack of intelligent means to assist. Most livestock farmers still conduct small-scale breeding in primitive ways, which is not conducive to the breeding and protection of Chengdu ma goats. In this paper, an automatic individual recognition method for Chengdu ma goats is proposed, which saves labor costs and does not depend on large-scale mechanized facilities. The main contributions of our work are as follows: (1) a new Chengdu ma goat dataset is built, which forms the basis for object detection and classification tasks; (2) an improved detection algorithm for Chengdu ma goats based on TPH-YOLOv5 is proposed, which is able to accurately localize goats in high-density scenes with severe scale variance of targets; (3) a classifier incorporating a self-supervised learning module is implemented to improve the classification performance without increasing the labeled data and inference computation overhead. Experiments show that our method is able to accurately recognize Chengdu ma goats in the actual indoor barn breeding environment, which lays the foundation for precision feeding based on sex and age.

## 1. Introduction

The Chengdu ma goat, the research object of this paper, has been listed in China’s National List of Livestock and Poultry Genetic Resources Protection. As an excellent local breed for both meat and skin in China, it has the advantages of fast growth, early maturity, high fecundity, strong adaptability, heat and humidity tolerance and stable genetic performance. Meanwhile, it is characterized by tender meat, no foul smell, a large skin area and excellent quality.

However, due to economic interests and the population’s low awareness of breed preservation, foreign breeds have been introduced for crossbreeding with Chengdu ma goats, resulting in a significant decrease in the proportion of purebred Chengdu ma goats, so the seed conservation of Chengdu ma goats is particularly important and urgent [1,2]. In addition, most sheep farmers still maintain a primitive and rough feeding method, which is not adapted to the needs of the development of modern animal husbandry.

The main reason that the sheep farming industry, represented by Chengdu ma goat farming, has failed to fully realize the transformation of agricultural modernization is that many farmers have no professional knowledge and their farming is only managed in a simple way. However, with the development and modernization of animal husbandry, it is obvious that such a breeding model is unable to meet the demand. Some notable problems are listed as follows:

Low reproduction rate. Due to the lack of scientific breeding concepts, farmers do not choose good-quality breeding rams for breeding. Moreover, inbreeding is common in the flock and no external good genes are passed on. This will lead to a lower reproduction rate and lower quality of the flock in the long run.

Inappropriate feed mix. Due to the lack of professional knowledge, farmers are unable to devise appropriate feeding programs to meet the developmental needs of breeding sheep according to the physiological characteristics and nutritional needs of rams and ewes, resulting in the lower productive potential and reproductive capacity of breeding sheep and a failure to prepare for the production of high-quality lambs, thus reducing the economic benefits for farms.

With the rapid development of information technology, combining artificial intelligence technology with animal husbandry can advance the development of animal husbandry in a more efficient and profitable way. Artificial intelligence is such a technology, which needs immediate implementation in the livestock industry. AI has emerged as a tool that empowers farmers in monitoring, forecasting and optimizing farm animal growth, which are some of the key areas in the livestock industry, where the use of artificial intelligence technology can yield rich dividends. As one of the representative innovations in the field of artificial intelligence, computer vision technology can be utilized in a large number of practical production environments, with its relatively manageable price and its non-contact, real-time and long-time working characteristics.

In recent years, the application of computer vision technology to the field of livestock production has attracted more and more attention. In 2018, Sarwar et al. combined deep learning algorithms and drone technology to count sheep using convolutional neural networks through video streams captured by drones [3], but their top-view angle image acquisition was mainly applied to free-range scenarios and could not be extended to sheep barn rearing. In 2019, Jwade et al. established a sheep image database containing four breeds and trained sheep breed classifiers based on deep learning methods to help shepherds to effectively distinguish different breeds of sheep [4], but their detection speed was slow and the method required the acquisition of fixed orientation images with the help of facilities to fix sheep bodies, which is only applicable to farms with a high degree of mechanization. In 2020, Xiang et al. demonstrated the counting and automatic tracking of sheep and cattle based on image and video data for application to intelligent farming systems [5]. In 2021, Sant’Ana et al. combined computer vision and machine learning techniques to predict the weight of sheep in a non-contact manner using images [6]. In 2022, Sant’Ana et al. finally used DenseNet201 to segment the background and individuals and classify gender in a superpixel dataset of sheep after comparing four different convolutional neural network architectures [7].

A synthesis of the above research work reveals that artificial intelligence technology represented by computer vision has brought innovation to the field of livestock production. However, some of the methods require a high degree of mechanization on the farm and require the assistance of other supporting facilities or have certain restrictions on environmental conditions, which do not have good universality and generalization ability. As the number of model parameters becomes increasingly large, the detection of livestock and poultry has obtained high accuracy, but the detection speed is correspondingly reduced. In practical applications, due to the storage space and power consumption limitations of the device, how to optimize the network structure to make the model lightweight and ensure the efficiency of detection is a key problem to be solved.

Deep learning-based methods mostly rely on supervised learning when applied to computer vision tasks. Supervised learning requires the use of large, manually labeled datasets for a specific task, and the heavy reliance on manual labeling leads to high labor costs and generalization errors [8], which is not conducive to portability and expansion. Therefore, it is important to eliminate the data dilemma so that the model can better learn the features of the image from the limited labeled data without increasing the labeling cost.

**Figure 1:**
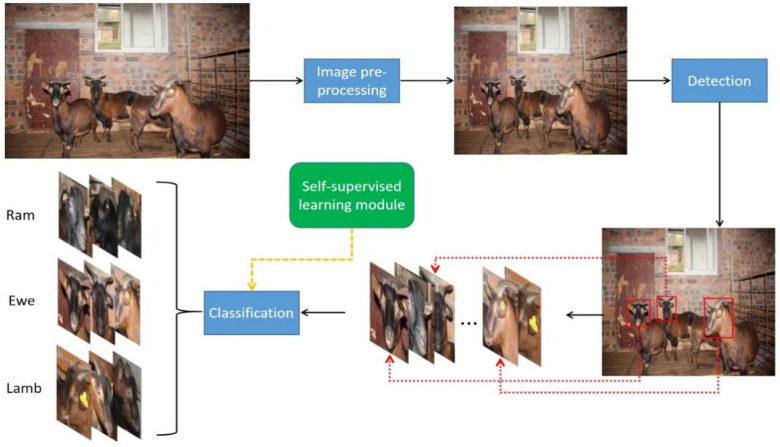
The overall pipeline of our framework. After preprocessing the images, the heads of Chengdu ma goats are detected using our improved object detection network. Since the classification effect of the detection network is not satisfactory, we construct a classifier incorporating a self-supervised module to accurately classify the detected heads.

To assist in the breeding of Chengdu ma goats based on the category information and make the subsequent intelligent tasks, such as precision feeding based on age and sex, possible, we use computer vision technology with deep learning to quickly and accurately identify the category of Chengdu ma goats based on two dimensions: sex and age. The living area of Chengdu ma goats is mainly concentrated in hilly and mountainous regions, which makes the deployment of large mechanized facilities difficult due to the vegetation cover and narrow terrain, and the data collection and detection in sheep barn scenarios cannot be performed by applying Unmanned Aerial Vehicles (UAVs) as in the literature [3]. Considering the special characteristics of the Chengdu ma goat breed and the complex feeding environment, we design a detection algorithm for goat head targets under the conditions of groups of dense goats in a barn and drastic changes in target scales. Moreover, a classifier for different classes of Chengdu ma goats is constructed, and the classification performance is improved by introducing a self-supervised learning module without increasing the inference time and additional labor cost of annotating. The overall pipeline is shown in Figure 1.

## 2. Related Work

### 2.1. Object Detection

The basic task of object detection is to use rectangular bounding boxes to determine the location and size of the detected objects in the image or video data, and it is necessary to determine the object class and give the corresponding confidence value [9]. With the rapid development of the deep learning techniques, the performance of the object detectors has been greatly improved.

The mainstream object detection algorithms based on deep learning can be divided into two categories: two-stage object detection algorithms and one-stage object detection algorithms [10]. Two-stage object detection algorithms, such as R-CNN [11], Fast R-CNN [12] and Faster R-CNN [13], first extract the region proposals from the image, and then perform secondary correction based on the region proposals to obtain the detection results. One-stage object detection algorithms such as YOLO [14], SSD [15] and RetinaNet [16] are based on a one-step framework of global regression and classification, which map directly from image pixels to bounding box coordinates and category probabilities, and can greatly improve the detection speed and better meet the demand for real-time detection.

#### 2.1.1. YOLO Series Object Detection Algorithms

You Only Look Once (YOLO) [14] is a well-known one-stage object detection algorithm, which is a milestone in the history of the development of one-stage detection. Its subsequent series of improved versions [17,18,19] have further improved the detection performance. At present, YOLOv5 is still one of the mainstream object detection algorithms and is widely used in various scenarios. YOLO consists of three main components:

(1) Backbone: A convolutional neural network is often used here to extract image features.
(2) Neck: A series of network layers that combine and reprocess image features and pass image features to the prediction layer.
(3) Head: It generates bounding boxes and prediction categories with corresponding confidence values. The confidence indicates the precision of the detection under specific conditions.

#### 2.1.2. TPH-YOLOv5

TPH-YOLOv5 [20] is an object detection algorithm based on YOLOv5, which is proposed mainly to solve the detection problem of the UAV capture scenario. The scene has challenges, such as drastic changes in the scale of the objects to be detected and dense objects, which are very similar to the scene of a sheep barn, and bring a great negative impact on the detection effect of the model.

TPH-YOLOv5 adds a detection head for tiny object detection. Combined with the other three detection heads, the four-detection-head structure can mitigate the negative effects of drastic changes in object scale. TPH-YOLOv5 uses Transformer Prediction Heads (TPH) for accurate object location in high-density scenes. To improve the detection performance of the model, TPH-YOLOv5 integrates CBAM [21] into the network to help the network to find regions of interest in images with large region coverage. Moreover, due to the poor classification results, a self-trained classifier is used to improve the classification ability for some easily confused categories.

### 2.2. Self-Supervised Learning

At a time when deep learning is rapidly advancing, it is inevitable that large-scale labeled data are needed to train deep neural networks in order to improve the performance of the models. However, the collection and labeling of large-scale datasets are not easy tasks and require expensive labor and time costs.

To reduce the substantial cost of collecting and labeling large-scale datasets, self-supervised learning, an important component of unsupervised learning methods, attempts to learn general image and video features from large-scale unlabeled data without using any manually labeled data [8]. Self-supervised learning mainly uses auxiliary tasks to mine supervised information from large-scale unsupervised data, and the network is trained with this constructed supervised information so that it can learn representations that are valuable for downstream tasks.

Designing efficient and task-appropriate assistive tasks is the key to self-supervised learning. Assistive tasks can be broadly classified into four major categories of methods: generation-based methods, contextual information-based methods, automatic semantic label generation-based methods and cross-modal methods. The self-supervised learning module used in this paper is related to auxiliary tasks based on contextual information, and the design of this class of methods mainly uses the contextual features of image or video data.

## 3. Materials and Methods

### 3.1. Data Acquisition

With the gradual reduction in Chengdu ma goat germplasm resources due to crossbreeding and other factors, only the Chengdu ma goat breeding farm of Chengdu Xilingxue Agricultural Development Co., Ltd. (Chengdu China) has been continuously committed to the conservation of their germplasm resources, and it was approved by the Ministry of Agriculture as a national Chengdu ma goat breeding farm in 2017. On the one hand, Xilingxue Agricultural Development Co., Ltd. has a large number of excellent purebred individuals. On the other hand, the breeding farm has standardized sheep barns with related facilities, which can truly reflect the real state of the sheep under standardized feeding conditions.

**Figure 2:**
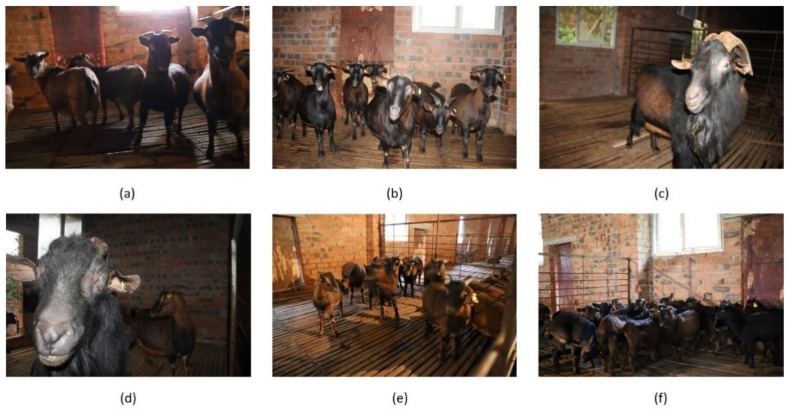
Some acquired images of Chengdu ma goats. It can be seen that the Chengdu ma goats are densely distributed in some of the images, and the body scale varies drastically in different images. (**a**): An image of ewes with poor lighting. (**b**): An image of ewes with good lighting. (**c**) An image of a ram with good lighting. (**d**) An image of a ram with poor lighting. (**e**) An image of lambs with good lighting. (**f**) An image of lambs with poor lighting.

We acquired images of Chengdu ma goats in the Chengdu ma goat breeding base of Chengdu Xilingxue Agricultural Development Co., Ltd. through manual shooting and camera monitoring. The breeding base is located in Dayi County, Chengdu City, Sichuan Province, China. This dataset was collected by Canon EOS 850D produced in Japan and EZVIZ C3WI wireless surveillance cameras produced in China. The shooting methods included long-distance shooting and short-distance shooting, and the shooting angle included top-down and parallel. The recording of the video included a top view and a forward view of the barns. The data included images and videos captured by the cameras under multiple time periods and lighting conditions, which covered single or multiple individuals and reflected the situation of the sheep house through multiple perspectives. Figure 2 shows some acquired images of Chengdu ma goats.

To reflect the actual living environment of the goats as much as possible by considering different light conditions, regional populations and sex ratio distributions, we selected 841 representative images from all of the images that were shot manually or extracted from the video as our experimental data. For the needs of the study, taking into account the practical application scenarios and animal characteristics, we labeled the Chengdu ma goats in the images with the help of professionals from two dimensions: sex and age. We labeled Chengdu ma goats as (adult) rams, (adult) ewes and lambs, and randomly divided the labeled images into a training set and test set according to the ratio of 7:3. Table 1 shows the information on the dataset used for our experiments.

### 3.2. Data Preprocessing

Deep learning usually requires a large amount of data to train the model. However, in reality, there are some data that are difficult to obtain, resulting in a small amount of data in this category, which does not meet the demand of data volume for deep learning. For this reason, experts have proposed data augmentation techniques to effectively solve this problem [22].

**Figure 3:**
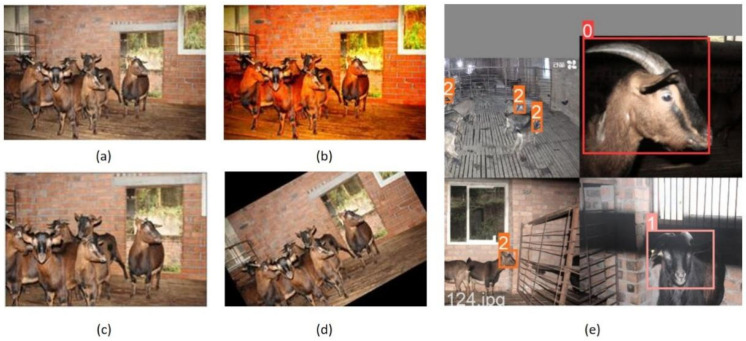
Some examples of data augmentation methods. (**a**): An original image. (**b**): Color dithering. (**c**): Equal scaling. (**d**): Rotation. (**e**): Mosaic, the multi-image fusion method. 0 represents ewe, 1 represents ram, and 2 represents lamb.

In the experiments, in addition to the conventional data augmentation methods such as geometric transformation and color transformation, a multi-image fusion method called mosaic was also used. Mosaic crops four images randomly and stitches them into a single image as training data. It has the advantage of enriching the background of the images and improving the batch size discretely, so that it can help to reduce the model’s dependence on a large batch size when training. Figure 3 shows some examples of data augmentation methods.

### 3.3. The Method for Chengdu Ma Goat Recognition

**Figure 4:**
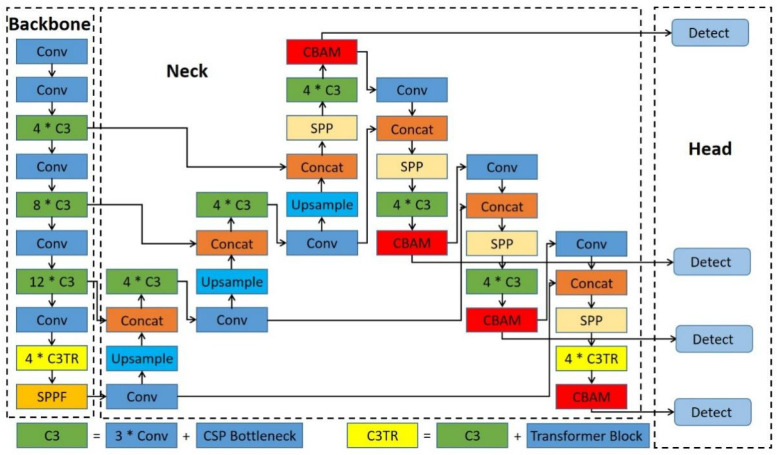
The architecture of the improved TPH-YOLOv5.

The dense distribution of Chengdu ma goats in some images, coupled with the severe variation in body scale from image to image, poses a great challenge to the effective detection of targets. In order to apply the model to the real-time detection task of Chengdu ma goats, the model needs to have good detection efficiency and deployability while addressing the above challenges with precise localization and classification. Therefore, two main improvements are made in this paper. The first are the improvements of the TPH-YOLOv5 architecture: based on the original TPH-YOLOv5 network, the number and position of transformer encoder blocks are adjusted to reduce the computational cost and make the model applicable to our task of detecting Chengdu ma goats while improving the detection speed; multiple SPP [23] modules are used to improve the scale invariance of images and enhance multi-scale feature fusion; BiFPN [24] is also introduced in the neck to enhance multi-level feature fusion and improve the network’s ability to learn and utilize features at different scales; and the numbers of CBAMs and some basic modules are adjusted according to the overall architecture of the network. The architecture of our improved TPH-YOLOv5 is shown in Figure 4. The second are the improvements of the classifier: a self-training classifier is constructed for Chengdu ma goat recognition. To avoid the large time and labor cost consumption caused by additional labeled data, a self-supervised learning module is introduced to learn the overall structure of the head and improve the accuracy of classification.

#### 3.3.1. The Improved TPH-YOLOv5

**Figure 5:**
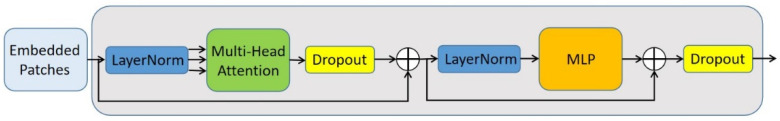
The architecture of a transformer encoder block, which contains two main sub-blocks. The first sub-block is a multi-head attention block and the second one (MLP) is a fully connected layer. Residual connections are used between each sub-block. Multi-head attention can help the current node to not only pay attention to the current pixels, but also obtain the semantics of the context.

TPH-YOLOv5 uses transformer encoder blocks to replace some convolution blocks and CSP bottleneck blocks in the original version of YOLOv5. The architecture of the transformer encoder is shown in Figure 5. Compared with the bottleneck block, the transformer encoder block can capture global information and richer contextual information [20]. Since TPH-YOLOv5 uses transformer encoder blocks after the backbone and before the four detection heads, it increases the computational overhead while improving the detection performance. Without affecting the detection effect of the model, we remove the transformer encoder blocks in front of the first three detection heads and keep only the transformer encoder blocks in front of the last detection head and at the end of the backbone, and we stack four transformer encoder blocks at these locations behind four C3 blocks, respectively. The input images of our model have high resolution and the feature maps at the end of the network have low resolution. Applying transformer encoder blocks on low-resolution feature maps can reduce the expensive computational overhead and memory cost.

**Figure 6:**
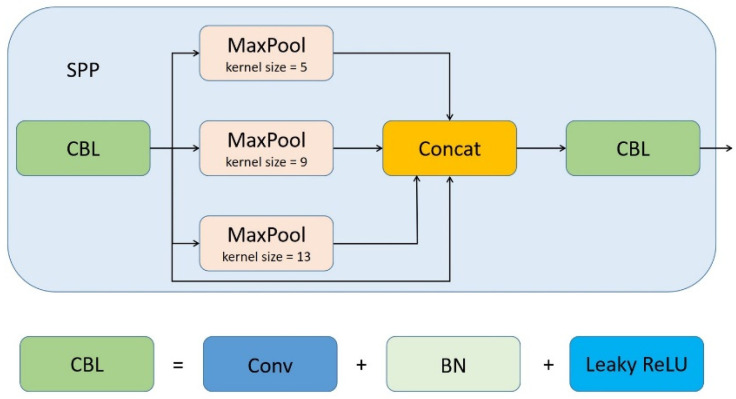
The architecture of SPP block in our improved TPH-YOLOv5.

The primary role of Spatial Pyramid Pooling (SPP) [23] is to solve the problem of the non-uniform size of the input images, and the fusion of different size features in SPP is beneficial in the case of large differences in object size in the detected images. TPH-YOLOv5 only uses the SPP block at the bottom of the backbone, and we add 4 SPP blocks in the neck of the network, which cause the feature maps to go through multiple multi-scale fusion steps, enriching the expression capability of the feature maps. In our network, SPP is implemented using kernels of uniform stride (stride = 1) but different sizes (5 × 5, 9 × 9 and 13 × 13), and feature fusion is achieved by channel stitching (concat). The architecture of SPP block is shown in Figure 6.

**Figure 7:**
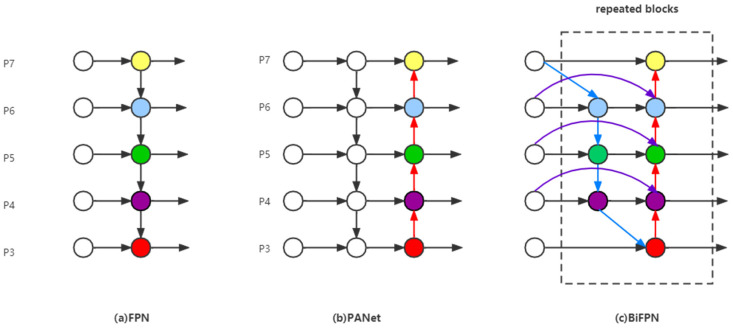
The architectures of three notable path aggregation networks. (**a**): FPN; (**b**): PANet; (**c**): BiFPN.

Generally, the features of shallow layers in the network have strong location information and are suitable for localization tasks; the features of deep layers in the networks have more advanced semantic information and are beneficial for classification tasks. FPN [25] combines positional and semantic information through the fusion of shallow and deep features, and predicts on different feature scales, so as to improve the detection performance. FPN has only top-down feature fusion, to which PANet [26] adds a bottom-up fusion path that more effectively utilizes the features extracted by the backbone. BiFPN [24] is a further improvement of the above path aggregation networks, which adds residual connections, removes edges with single input nodes to reduce the computational effort and uses a weighting mechanism for each scale feature to be fused to adjust the contribution of each scale. The structures of FPN, PANet and BiFPN are shown in Figure 7. By replacing PANet with BiFPN in TPH-YOLOv5, a better trade-off between accuracy and efficiency can be achieved.

#### 3.3.2. The Classifier Incorporating a Self-Supervised Learning Module

After TPH-YOLOv5 was trained on the VisDrone2021 dataset, it was tested and found to have good localization ability and relatively weak classification performance. Therefore, the authors constructed a training set by cropping the ground-truth bounding boxes and resizing each image patch to 64 × 64, and then selected ResNet18 [27] as the self-trained classifier, which resulted in a small improvement in accuracy [20].

In the Chengdu ma goat recognition task, some adult ewes and lambs outwardly expressed minor differences in physical characteristics, with both body coat colors showing russet or brown [28], while both horns were short and mostly sickle-shaped. The self-trained classifier mentioned in the paper of TPH-YOLOv5 uses only conventional means to learn image features and enhance the classification performance, which cannot well solve the above-mentioned problem in the task of Chengdu ma goat classification.

At present, most recognition methods based on deep learning are committed to learning the external and significant representative features of the target object, while ignoring the overall structure of the object, which also leads to the traditional methods based on deep learning yielding incorrect results in many cases, such as distinguishing the front and rear wheels of a car and locating the legs of a bird in the branches [29]. While extracting the external features of the object is important, learning the overall structure of the object is also crucial. It is extremely challenging to learn the overall structure of an object systematically without adding additional annotations and without increasing the inference time.

Look-into-Object (LIO) [29] understands the structure of objects in images by automatically modeling contextual information between regions. LIO explores the process of object recognition from a psychological perspective, dividing object recognition into two steps, first roughly determining the overall range of the recognized object and subsequently analyzing the internal structure of the object, according to which two modules are designed. The first one is the Object-Extent Learning (OEL) module for localizing the overall range of objects, and the second one is the Spatial Context Learning (SCL) module for modeling the internal structure of objects.

Spatial Context Learning (SCL) aims to model the location relationships of different parts of the target region by predicting the polar coordinates of the non-central part of the target region with respect to the central part of the target region (the origin of the polar coordinates). The SCL module uses polar coordinates to measure the spatial connection between different regions. Because the targets exist in different forms, modeling the position relationship between different parts using the Cartesian coordinate system requires modeling the absolute position, which is sensitive to the reference coordinate system and can be difficult to model. The polar coordinate system does not have this problem, as it uses relative coordinates; all positions are calculated relative to the pole, and modeling is relatively easy. The SCL module takes the relative distance differences and polar angle differences as the loss, so that the backbone network can recognize the composition of the object.

**Figure 8:**
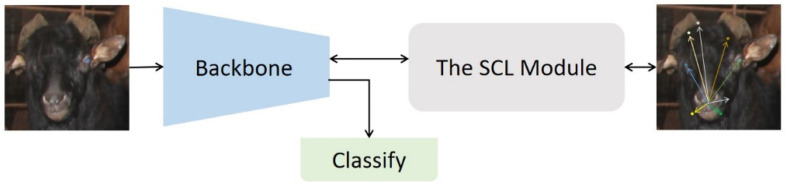
Spatial Context Learning. The SCL module forces the backbone to learn the internal structure of the object.

In the sheep barn scenario, sheep are densely packed, and there is often overlap between sheep bodies, so it is not easy to use sheep bodies as recognition information. The SCL, as a self-supervised module, can enhance the ability of the backbone network to extract the overall structural information of the image by learning the spatial contextual relationship of the head region of the sheep without bringing an additional manual labeling cost. Figure 8 shows how the SCL module works on the network.

ResNet [27] uses residual blocks to effectively alleviate the model degradation problem of deep networks. As a classical feature extraction network, it has been widely applied in academic research. ResNeXt [30] introduces the idea of aggregated transformations on the basis of ResNet by stacking blocks of the same topology in parallel, which improves the classification performance without increasing the parameter order of magnitude, and also effectively controls the number of hyperparameters.

We use ResNeXt50 as the backbone network and incorporate the SCL module to construct a classifier for Chengdu ma goats. The feature maps extracted from the backbone network are further fed to the SCL module when training. After end-to-end training, the backpropagation signal from the SCL module can optimize the representation learning of the backbone network. In this way, structural information can be injected into the backbone network to improve object recognition without requiring additional annotation information. In addition, the SCL module can be disabled during the inference time, thus not increasing the computational overhead during inference.

#### 3.3.3. Implementation Details

We implement all the algorithms using Python 3.8 and Pytorch 1.9.0. All models were trained and tested using an NVIDIA RTX3090 GPU. We used SGD as the optimizer for training. For the object detection models, the initial learning rate was set to 0.01, the momentum was 0.937, the weight decay coefficient was 0.0005, the batch size was 8, and the epoch was 80. For the self-trained classifiers, the learning rate was set to 0.0001, the momentum was 0.9, the batch size was 8, and the epoch was 100.

## 4. Results

### 4.1. Detection

**Figure 9:**
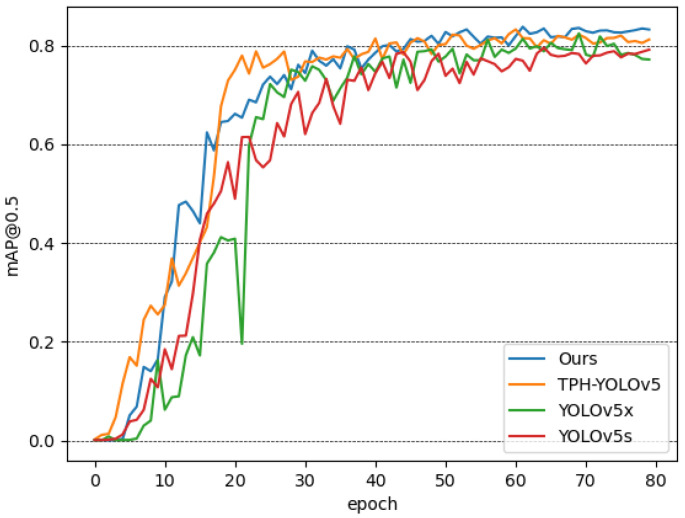
The mAP of the detectors in the test set.

YOLOv5 can be divided into four versions according to the model size, including YOLOv5s, YOLOv5m, YOLOv5l and YOLOv5x. The number of parameters, computation and detection performance of these four versions are in increasing order, with YOLOv5s having the lightest network structure and YOLOv5x having the best detection performance. Figure 9 shows the mAP of the detectors in the test set.

As shown in Table 2, although the computation of YOLOv5s is small, it has a relatively low mean average precision (mAP), which means that its detection effect is not good enough. Our detector has similar computational effort as YOLOv5x, but is faster to train and has a 1.96% improvement in mAP. Compared with the original TPH-YOLOv5, our detector improves the mAP by 1.57%, the computational effort is reduced, and the training time is shortened. The experiments verify the effectiveness of our method.

To further prove the effectiveness of our improvement, the results of the ablation study are shown in Table 3. The ablation study showed that SPP and BiFPN can indeed improve the mAP, but they also increase the inference time. Transformer encoder blocks have large computational overhead, so we tried applying them only at the end of the network, with low-resolution feature maps. The ablation study showed that for the Chengdu ma goat detection task, removing the transformer encoder blocks in front of the first three detection heads hardly affects the mAP, but significantly shortens the inference time, which proves that our attempt is sensible.

### 4.2. Classification

**Figure 10:**
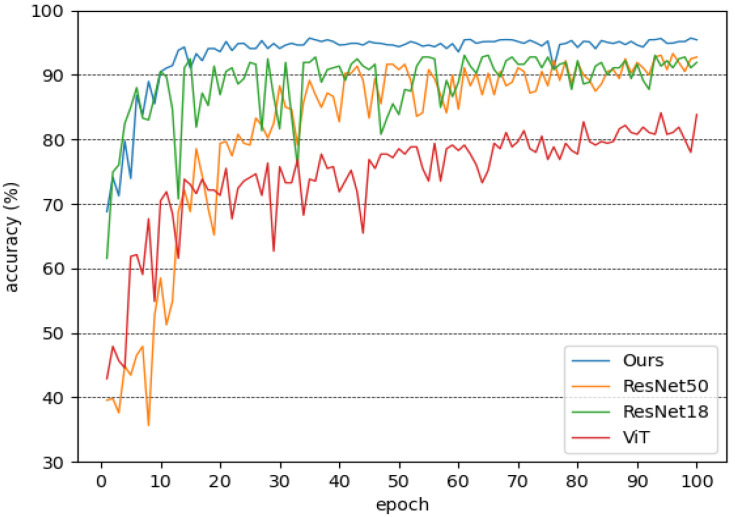
Accuracy of the classifiers in the test set.

The classifier plays an important role in our overall pipeline. The classifier proposed in this paper is ResNeXt50, incorporating a self-supervised learning module. In the experiments, we compared our constructed classifier with several mainstream classifiers, including ResNet and ViT [31]. Figure 10 shows the accuracy of the classifiers in the test set.

ViT lacks some of the inductive biases inherent to convolutional neural networks, such as translation equivariance and locality [31], and therefore does not generalize well on a small-scale dataset such as our Chengdu ma goat dataset. ResNet50 has good comprehensive performance and has a wide range of applications in various computer vision tasks. ResNeXt50 is a variant of ResNet50; we use ResNeXt50 (with 0.3% accuracy improvement compared to using ResNet50) as the backbone network of the classifier for Chengdu ma goats, and incorporate a self-supervised learning module, using the idea of spatial context learning to reinforce the backbone network to learn the internal structure information of Chengdu ma goat images. As shown in Table 4, in the task of Chengdu ma goat classification, the accuracy of the classifier proposed in this paper is higher than that of other representative classifiers, up to 95.70%.

**Figure 11:**
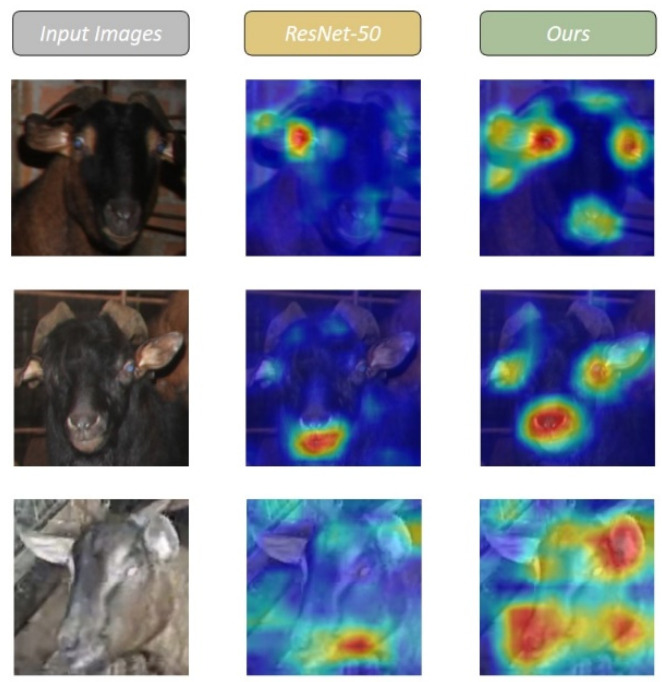
Feature map visualization based on the last convolutional layer of the backbone. The first column shows the original images, while the second and the third columns show the maximally responding feature maps from the ResNet50 and our classifier, respectively.

To help understand and analyze the working principle and decision process of the classifier for Chengdu ma goats proposed in this paper, we generated class activation mapping images using Grad-CAM [32]. As shown in Figure 11, in most cases, regardless of the differences in sex and age, the proposed classifier in this paper better mines the overall structural information of the head images, which facilitates accurate classification.

## 5. Discussion

A review of the literature shows that artificial intelligence technology has been increasingly applied to livestock production in recent years, but limitations still exist. The authors in [3] explore the combination of deep learning algorithms and UAV technology applied to intelligent sheep farming, which is only applicable to the free-range scenario. The authors in [4] explore the use of deep learning algorithms for sheep breed classification, with shortcomings such as slow detection speed and dependence on the facility for fixing sheep bodies. In addition, there are few recognition methods especially for the specific group of Chengdu ma goats, and there is still some room for exploration and improvement.

In this paper, we take the Chengdu ma goat as the research object and discuss the method for Chengdu ma goat recognition in barns. We improve TPH-YOLOv5 to build our detector. Compared with YOLOv5s, with the highest detection efficiency, and YOLOv5x, with the best detection effect in YOLOv5, our detector obtains the best mAP of 83.78% with similar training time. The mAP of our detector even exceeds that of the original TPH-YOLOv5, while our detector needs less computational overhead and a shorter training time. Our experiments demonstrate the impressive and comprehensive performance of our improved detector for detecting Chengdu ma goats in densely packed conditions with large scale variance of targets. Meanwhile, comparing representative classifiers of different architectures, the classifier built by introducing a self-supervised learning module demonstrates effectiveness in improving the classification performance for Chengdu ma goats by learning the structure of the head region when trained on very limited amounts of labeled data.

In the head image classification experiments, ViT, representing the transformer architecture, gives poor classification results. With the expansion of the data volume, further research can be attempted to fully exploit its potential learning ability on image features. Our classifier with the self-supervised module works well for classifying specific sheep species and can further learn more generalized features to solve more sheep species recognition problems.

## 6. Conclusions

In this paper, a Chengdu ma goat image dataset for computer vision tasks is constructed. Precise detection of Chengdu ma goats is challenging due to the dense distribution of the targets in sheep barns and the large differences in body scale in different images. By improving the structure of TPH-YOLOv5 to build a detector and constructing a classifier incorporating a self-supervised module with ResNeXt50 as the backbone network, we propose a general framework for Chengdu ma goat recognition, which provides a new idea for solving Chengdu ma goat recognition problems in barns. The experiments verify the effectiveness of our method. In addition to improving the performance while taking into account the usability of the model, implementing functions such as abnormal behavior detection of livestock will be our subsequent work. As more types and increased quantities of livestock data become available, we will apply our models to a wider range of areas to help develop intelligent technology in animal husbandry. In the near future, automated precision feeding of livestock will become possible with the aid of our method.

## Figures and Tables

**Table 1:** Information on the dataset used for our experiments.

Class	The Number of Labeled Goats in the Training Set	The Number of Labeled Goats in the Test Set	Total
Ewe	1033	289	1322
Ram	136	38	174
Lamb	896	604	1500
All	2065	931	2996

**Table 2:** The comparison of several detectors in our experiments.

Methods	mAP@0.5 (%)	GFLOPs	Training Time (Hours)	Inference Time (ms)
YOLOv5s	79.55	16.4	1.612	17.4
YOLOv5x	81.82	217.4	2.205	23.2
TPH-YOLOv5	82.21	556.8	3.279	33.2
Ours	83.78	270.4	1.854	27.6

**Table 3:** Ablation study on our dataset. TPH-YOLOv5+C3TR denotes that TPH-YOLOv5 uses transformer encoder blocks after the backbone and before the last detection head only, removing the transformer encoder blocks in front of the first three detection heads.

Methods	mAP@0.5 (%)	Inference Time (ms)	
TPH-YOLOv5	82.21	33.2	
TPH-YOLOv5 + SPP	82.97	34.6	
TPH-YOLOv5 + BiFPN	83.24	35.9	
TPH-YOLOv5 + C3TR	82.12	24.1	
TPH-YOLOv5 + C3TR + SPP + BiFPN	83.78	27.6	

**Table 4:** The comparison of several classifiers in our experiments.

Methods	Accuracy (%)
ViT	84.12
ResNet18	93.04
ResNet50	93.31
Ours	95.70

## Data Availability

The data used to support the findings of this study are available from the corresponding author upon request.
